# Retinal Macroglial Responses in Health and Disease

**DOI:** 10.1155/2016/2954721

**Published:** 2016-05-18

**Authors:** Rosa de Hoz, Blanca Rojas, Ana I. Ramírez, Juan J. Salazar, Beatriz I. Gallego, Alberto Triviño, José M. Ramírez

**Affiliations:** ^1^Instituto de Investigaciones Oftalmológicas Ramón Castroviejo, Universidad Complutense de Madrid, 28040 Madrid, Spain; ^2^Departamento de Oftalmología y ORL, Facultad de Óptica y Optometría, Universidad Complutense de Madrid, 28037 Madrid, Spain; ^3^Departamento de Oftalmología y ORL, Facultad de Medicina, Universidad Complutense de Madrid, 28040 Madrid, Spain

## Abstract

Due to their permanent and close proximity to neurons, glial cells perform essential tasks for the normal physiology of the retina. Astrocytes and Müller cells (retinal macroglia) provide physical support to neurons and supplement them with several metabolites and growth factors. Macroglia are involved in maintaining the homeostasis of extracellular ions and neurotransmitters, are essential for information processing in neural circuits, participate in retinal glucose metabolism and in removing metabolic waste products, regulate local blood flow, induce the blood-retinal barrier (BRB), play fundamental roles in local immune response, and protect neurons from oxidative damage. In response to polyetiological insults, glia cells react with a process called reactive gliosis, seeking to maintain retinal homeostasis. When malfunctioning, macroglial cells can become primary pathogenic elements. A reactive gliosis has been described in different retinal pathologies, including age-related macular degeneration (AMD), diabetes, glaucoma, retinal detachment, or retinitis pigmentosa. A better understanding of the dual, neuroprotective, or cytotoxic effect of macroglial involvement in retinal pathologies would help in treating the physiopathology of these diseases. The extensive participation of the macroglia in retinal diseases points to these cells as innovative targets for new drug therapies.

## 1. Introduction 

The macroglial cells of the retina are the astrocytes and the Müller cells. Macroglial cells perform various essential roles for the normal physiology of the retina, maintaining a close and permanent relationship with the neurons [1]. Under normal conditions, the retinal macroglia provide trophic and metabolic support to neurons and are responsible for maintaining the homeostatic environment required for appropriate neuronal functioning. Furthermore, they are involved in the formation of the BRB and might even play a role in the correct transmission of nerve impulses [2]. Glia, as a population of immune cells residing in the retina and the optic nerve, are able to respond and become activated rapidly in the presence of any type of damage, in order to safeguard the immune privilege of nervous tissue [3]. Reactive gliosis has a direct neuroprotective effect on the retina. By contrast, chronic gliosis exacerbates disease progression, increasing vascular permeability, infiltration of toxic compounds, and even neovascularization [4].

The aforementioned data underline the importance of undertaking studies aimed at improving our understanding of the role of macroglia in the pathogenesis of retinal diseases. Such knowledge could help to develop novel neuroprotective therapies for medical treatment of these diseases.

## 2. Glial Cells

Glial cells have long been considered purely passive elements within the nervous system. Yet, their proximity to the neurons and blood vessels involves them in vital tasks that are essential for neuronal survival [5]. Glial cells are subdivided into macroglial cells (astrocytes and oligodendrocytes) and microglial cells. Astrocytes represent the most abundant and morphologically heterogeneous neuroglial cell, these including protoplasmic astrocytes, fibrous astrocytes, and radial glia (Bergmann glia of the cerebellum and Müller cells of the retina) [6, 7]. Oligodendroglia are responsible for myelination and metabolic support of the axon, while astrocytes are more involved as key players in neuronal circuits, information processing, and maintenance of synaptic integrity [8, 9].

## 3. Retinal Macroglia

Overall, in the vascular retina of many vertebrates (including mammals) two basic types of macroglial cells are found: Müller cells and astrocytes. The oligodendrocytes are seen occasionally in the retina, but only when myelinated ganglion cell axons are present in the nerve-fiber layer [1, 10–15]. Müller cells are long, radially oriented cells, which span the width of the neural retina from the outer limiting membrane (OLM), where their apical ends are located, to the inner limiting membrane (INL), where their basal end feet terminate. Müller cells ensheath all retinal neural somas and processes. Each of these cells can be considered the core of a columnar microunit of retinal neurons [16, 17]. Thus, Müller cells constitute an anatomical link between the retinal neurons and the compartments with which these need to exchange molecules (the retinal blood vessels, vitreous body, and subretinal space) [18].

**Figure 1:**
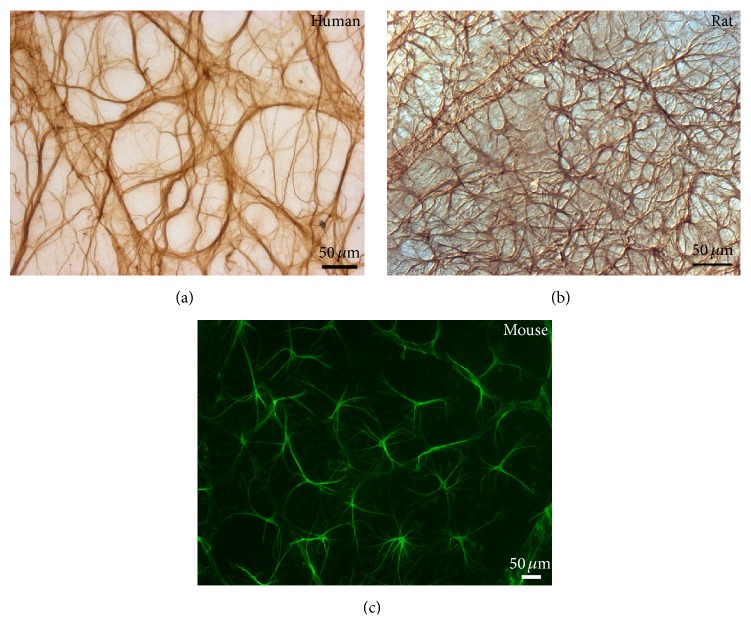
Retinal astrocytes. Retinal whole-mount. Immunoperoxidase ((a) and (b)). Immunofluorescence (c). Astrocytes in the normal retina of a 58-year-old man. In the ganglion cell layer of the human retina, star-shaped astrocytes form a honeycomb plexus (a). In the rat (b) and mouse (c) retina star-shaped astrocytes form a plexus distributed throughout the retina. Such plexus is denser in the rat than in the mouse retina.

Retinal astrocytes are located mainly in the nerve-fiber layer (NFL) and ganglion cell layer (GCL) in most mammals, that is, human, rats, and mouse [1, 19, 154]. In rabbits, astrocytes are confined to the medullary nerve-fiber region, which is the only vascularized area in the rabbit retina [13]. Retinal astrocyte morphology differs between species. In humans, two types of astrocytes can be distinguished: elongated astrocytes (located in the NFL) and star-shaped astrocytes (located in the GCL) (Figure 1(a)) [12, 20]. In mice and rats the astrocytes are star-shaped (Figures 1(b) and 1(c)) [19, 20].

Macroglial cells are permanently in close relationship with neurons, performing various essential roles for the normal physiology of the retina [1, 12–14]. Thus, every aspect of the development, homeostasis, and function of the visual system involves a neuron-glia partnership. Unlike retinal ganglion cells (RGCs), astrocytes do not propagate action potentials along their processes; however, astrocytes and Müller cells do exhibit regulated increases in intracellular calcium concentrations [Ca^2+^]i that represent a form of astrocyte excitability [21–24]. Increases in astrocytic [Ca^2+^]i are of functional significance in astrocyte/astrocyte and astrocyte/neuron communication.

**Figure 2:**
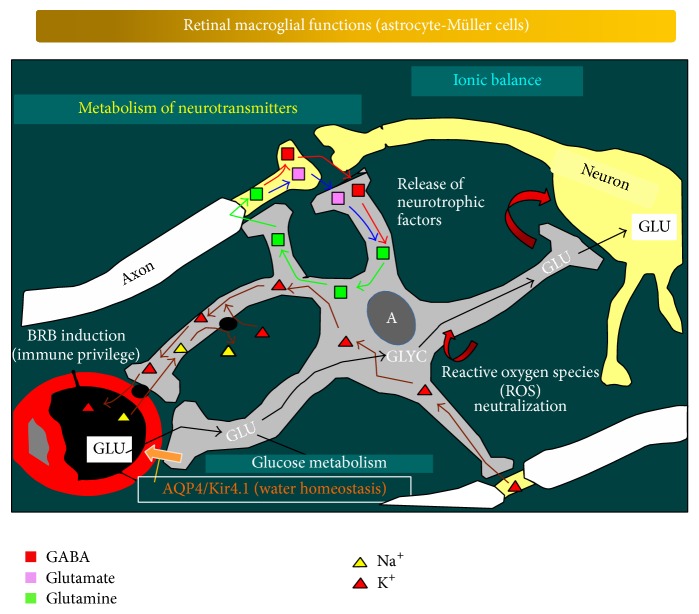
Schematic drawing illustrating the functions of the retinal macroglia. Macroglial cells perform various essential roles for the normal physiology of the retina, maintaining a close and permanent relationship with the neurons. The scheme illustrates the links between the retinal macroglia, the neurons, and the blood-retinal barrier (from [154]).

Astrocytes and Müller cells participate in the structural organization of the retina through the creation of nonoverlapping microanatomical domains that integrate into macroglial syncytia through gap junctions [12]. This organization allows long-distance communication within glial networks [25]. Macroglial cells insulate neurons, provide physical support for them, and supplement them with several metabolites and growth factors (Figure 2). These cells are also important in axon guidance and in the control of synaptogenesis [26, 27] and can adopt stem-cell properties [28, 29].

Under normal conditions, astrocytes and Müller cells maintain the homeostasis of extracellular ions and other metabolites, water, and pH (Figure 2). It has been demonstrated that a complex glutamatergic-purinergic signaling cascade enables Müller cells to maintain their cell volume. Tight cell-volume regulation is a prerequisite for Müller cells to mediate transcellular ion and fluid fluxes from the extracellular space of the retina to reservoirs such as the vitreous body or blood vessels, thus in turn enabling the spatial buffering of potassium and the maintenance of retinal-fluid homeostasis [7, 30]. In addition, astrocytes and Müller cells are involved in maintaining the homeostasis of neurotransmitters such as glutamate and GABA (Figure 2) [31]. After the reuptake of neurotransmitters into astrocytes, the neurotransmitters are metabolized and transformed into precursors that can be returned to the neurons to be converted into active neurotransmitters. The astrocytes directly interact with neurons during synaptic activity in a manner that is essential for information processing in neural circuits. Such evidence has given rise to the “tripartite synapse” hypothesis [32–34]. The synapses in the CNS appear to be constituted by three elements: the perisynaptic astroglial processes, the presynaptic neuron, and the postsynaptic one [34]. In this architecture, astrocytes have a dual role. These cells in fact can sense the transmitter release as they express many neurotransmitter receptors, and, on the other hand, astrocytes can modulate the efficacy of the synapse by releasing gliotransmitters (i.e., glutamate, GABA, ATP, and D-serine), thus accurately modulating synaptic transmission [35].

Macroglial cells are also involved in retinal glucose metabolism (Figure 2), providing retinal neurons with nutrients such as lactate/pyruvate for their oxidative metabolism [18, 36, 37] and in removing metabolic waste products. These cells also produce a great quantity of cytokines and growth factors [38, 39], which may contribute to both neurotoxic and neuroprotective effects [40]. In addition, they produce laminin, fibronectin, and tropoelastin, the precursor of elastin [39]. Astrocytes and Müller cells have also been demonstrated to be more resistant to oxidative damage than neurons; this characteristic protects them against such damage. This potential is due to the fact that these cells contain high concentrations of antioxidants such as reduced glutathione and vitamins (Figure 2) [41]. Reduced glutathione is provided to neurons [42, 43] and acts as a scavenger of free radicals and reactive oxygen compounds [18]. Another way of neuroprotection is the uptake and/or detoxification of potentially harmful substances and even particles (either intrinsic or foreign). This involves the phagocytosis of debris from death neurons or pigment epithelial cells [44–48]. Consequently, depression of these cellular activities could lead to neuronal dysfunction [49].

Astrocyte and Müller cells are involved in regulating local blood flow [29] in response to changes in neuronal activity [50]. Indeed, a number of molecules, such as prostaglandins (PGE), nitric oxide (NO), and arachidonic acid (AA), which increase or decrease CNS blood-vessel diameter and blood flow, are produced by astrocytes [51, 52]. Astrocytes and Müller cells induce the properties of the barriers in the retinal capillaries, the BRB (Figure 2) [53, 54]. They release substances that stabilize the tight junctions between endothelial vascular cells [54], securing immune privilege to protect neurons from the potential damage of an inflammatory immune response (Figure 2). In addition, glial cells play fundamental roles in local immune responses and immunosurveillance [55, 56].

Finally, Müller cells act as “light guides.” Their orientation and low scattering make these cells able, like optical fibers, to conduct the light into the interior of the retina to fall on the photoreceptors with less degradation [57].

### 3.1. Astrocytes and Retinal Diseases

As mentioned above, the main function of astrocytes is to maintain the homeostasis of the nervous tissue and to control, protect, and support neuronal function [58]. Astroglial cells defend the CNS and therefore the retina from damage through a process called reactive gliosis. This gliosis is triggered in response to polyetiological insults [59] such as trauma, ischemic damage, neuroinflammation, or neurodegeneration. This response seeks to maintain retinal homeostasis involving both morphological and functional alteration in the glial cells [60]; however, when malfunctioning, astroglia can also constitute the primary pathogenic element [61].

Reactive astrogliosis is an evolutionarily conserved defense program, which is disease- and context-specific and involves the activation of thousands of genes [59, 61]. Thus, at least 50% of the injury-altered gene expression is injury-type specific [62].

The hallmarks of reactive astrogliosis are a burst in astrocyte number (hyperplasia/proliferation), increased number and length of astroglial processes, larger cell body size (hypertrophy), migration, and upregulation of cytoskeletal components such as glial fibrillary acidic protein (GFAP), vimentin, and nestin [45, 47, 48, 59, 61, 63]. The deletion of GFAP and vimentin genes in a genetic mice model of Alzheimer's disease (AD)* in vivo *resulted in a complete inhibition of astroglial activation [64]. The increased expressions of these intermediate filaments are, however, considered only as broad markers of this process, because astrogliotic metamorphosis may produce many different, yet to be fully characterized, reactive phenotypes specific to different diseases.

A reactive astrogliosis has been reported in different retinal pathologies. In AMD a large number of reactive and hypertrophic astrocytes have been found [47]. In experimental diabetic retinopathy the glial reactivity was manifested by increased GFAP immunoreactivity and content in astrocytes [65]. In the final stages of retinitis pigmentosa, when the ganglion cells disappear, the only cells left are reactive hyperplasic astrocytes [66]. In both the human glaucomatous optic nerve head and the retina of different animal models of glaucoma, greater GFAP expression has been detected [19, 67, 68]. In a mouse model of laser-induced ocular hypertension (OHT), both contralateral and OHT eyes have intensified GFAP immunoreactivity with respect to the naïve animals; the retinal macroglia of contralateral normotensive eyes exhibited morphological signs of reactivity that differed from naïve and OHT eyes. Astrocytes in contralateral eyes were more robust and had an increase in GFAP-labeled retinal area in comparison to naïve ones, although astrocytes in OHT eyes showed fewer secondary processes and a reduction in the GFAP-labeled retinal area with respect to contralateral and naïve eyes [19]. In addition, it has been noted that astrocytes proliferated at the optic nerve head and in the lateral geniculate nucleus and visual cortex in human glaucoma and animal glaucoma models [69, 70], postulating that astrocytosis is key in the remodeling of the optic nerve head during glaucomatous damage [71].

**Figure 3:**
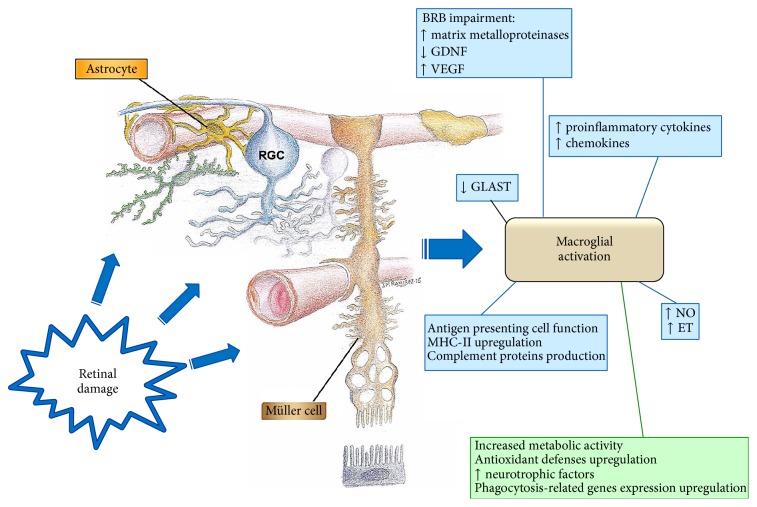
Diagram summarizing the functions of activated macroglia. Under conditions of tissue stress that might represent a risk to neuronal survival, glial cells undergo reactive gliosis. The aim of acute gliosis is to protect the nervous tissue by reestablishing the extracellular medium and by supplementing neurons with factors that promote their survival (in green). An uncontrolled response (in blue), as occurs in most neurodegenerative diseases, will harm the tissue [BRB: blood-retinal barrier; ET: endothelin; GDNF: glial cell derived neurotrophic factor; GLAST: glutamate aspartate transporter; MHC: major histocompatibility complex; NO: nitric oxide; RGC: retinal ganglion cell; VEGF: vascular endothelium growth factor].

In the processes of a productive gliotic response, astrocytes undergo complex remodeling of their biochemistry and function, which generally leads to neuroprotection (Figure 3) [59]. A growing group of studies evidence a beneficial role for activated astrocytes in neuroinflammation associated with neurodegenerative diseases [72]. Activated astrocytes stimulate higher metabolic activity, increase the expression of cytoprotective factors, and restore neurotransmitter balance and ion and water concentration, among other benefits [20, 45–47, 63]. This reactive gliosis has been associated with the upregulation of enzymatic and nonenzymatic antioxidant defenses that may fortify the ability of the astrocytes to protect neurons from free radicals (Figure 3) [41]. In an experimental model of glaucoma in rats the retinal area occupied by astrocytes in eyes diminished with ocular hypertension; this trend is stronger in eyes with higher levels of intraocular pressure [20]. The authors postulate that, in RGC, death would start when astrocytes fell below a specific level, and thus a minimal amount of retina covered by astrocytes could be necessary to protect the RGC [20].

Astrocytes provide neurotrophic factors for RGC support; this is particularly important in glaucoma in which blockage in the axonal transport inherent to this pathology can impair neurotrophin delivery from the visual pathway, such as the superior colliculus. The loss of astrocytes in the lamina cribrosa during glaucomatous neurodegeneration could compromise RGC survival [73].

During the CNS injury, astrocytes become reactive and migrate to the damage site where they isolate the injured area and remove pathogens, dying cells, and cellular debris and then remodel the nerve tissue on resolving the pathology (Figure 3) [61]. In AMD, hypertrophic and reactive astrocytes have been observed to phagocytize the residues of ganglion cells that have died through necrosis or apoptosis [48]. Astrocytes in glaucoma have shown an upregulated expression of the phagocytosis-related gen Mac-2, in the laminar and orbital region of the optic nerve, suggesting that astrocytes could participate in the clearance of the RCG axonal debris [74].

As noted above, reactive gliosis includes the onset of signaling mechanisms that are primarily protective for retinal neurons but may proceed uncontrolled to augment neuronal damage [40, 75]. Chronic gliosis is typically injurious, directly and indirectly damaging neurons and the vasculature while also inhibiting tissue repair [76]. During chronic diseases such as angiogenic vascular conditions in the eye (diabetic retinopathy, retinal-vein occlusion, retinopathy of prematurity, and AMD), reactive astroglia through vascular endothelial growth factor (VEGF) production exacerbate disease progression, increasing vascular permeability and even neovascularization [4]. Also, reactive astroglia produce molecules that inhibit axon regeneration and repair, triggering neurocytotoxicity or secondary damage in nearby neurons and glial cells [59, 77, 78]. The absence of GFAP and vimentin reportedly attenuates retinal-detachment-induced reactive gliosis and subsequently limits photoreceptor degeneration [79]. Moreover, the inhibition of reactive gliosis prevents apoptotic death of retinal neurons and provides substantial neuroprotection [80].

Although the microglia cells are the main mediators in the inflammatory damage of the CNS during neurodegeneration, astrocytes behave similarly to microglia and together can act synergistically, promoting chronic neuroinflammation or fostering neuroprotection [72]. Most inflammatory mediators produced by astrocytes may act on microglial cells, thus facilitating chronic microglial activation and thereby favoring neuronal death [81]. Similarly, inflammatory mediators produced by microglia may intensify astrocyte activation [82, 83]. TNF-*α* promotes the synthesis and release of glutamate in microglia and glutamate uptake in astrocytes, both mechanisms augmenting neuronal loss [72, 84]. It has been shown that high extracellular levels of TNF-*α* exacerbate the inflammation and neurodegeneration mediated by astrocytes. However, low levels of TNF-*α* secreted mainly by astrocytes autocrinely stimulate the secretion of neurotrophic factors, supporting neuronal survival [85]. In an experimental model of glaucoma in rats, Lee et al. (2014) suggested that TNF-*α* released by activated microglia stimulated macroglial cells to produce neuroprotective factors, including nerve-growth factor, in response to a mid-hypertensive glaucomatous injury [86].

In both experimental and human glaucoma, in addition to TNF-*α*, astrocytes can produce and/or respond to other neurotoxic molecules such as NO, IL-6, and endothelins (ETs) which could directly damage RCG axons (Figure 3) [87–89]. ETs are potent vasoconstrictive molecules that are produced by astrocytes and act in a paracrine loop on ET receptors to trigger astrocyte activation and proliferation and to impede ocular blood circulation [90]. The expression of ET-1 receptors (ETA and ETB) has been described both in human and in experimental glaucoma [91]. In addition, the overexpression of ET-1 in the optic nerve head correlated with neural loss in an experimental model of glaucoma [92, 93].

Reactive astrocytes can secrete inflammatory cytokines, such as IL-1*β*, IL-6, and IL-8 (Figure 3) [94]. In diabetic retinopathy, hyperglycemia boosts astrocyte cytokine expression, activating NF-*κ*B and intensifying oxidative stress [95]. After experimental retinal detachment, astrocytes become a major source of IL-1 production in the neural retina [96].

Apart from inflammatory cytokines reactive astrocytes can secrete chemokines, including CCL2, CCL5, CCL20, CXCL10, CXCL12, CXCL1, CXCL2, and CX3CL1 (Figure 3) [97]. These chemokines are involved in the recruitment of microglia, monocytes/macrophages, T-cells, and dendritic cells at the inflamed sites of the CNS [72]. Furthermore, the inflammatory mediators secreted by reactive astrocytes could affect the properties of the blood-brain barrier (BBB), thereby facilitating the infiltration of peripheral immune cells within the brain parenchyma during neurodegenerative diseases [98, 99]. Macroglial dysfunction in rats results in BRB breakdown of retinal vascular diseases through reduced expression of the tight-junction protein claudin-5 [100].

**Figure 4:**
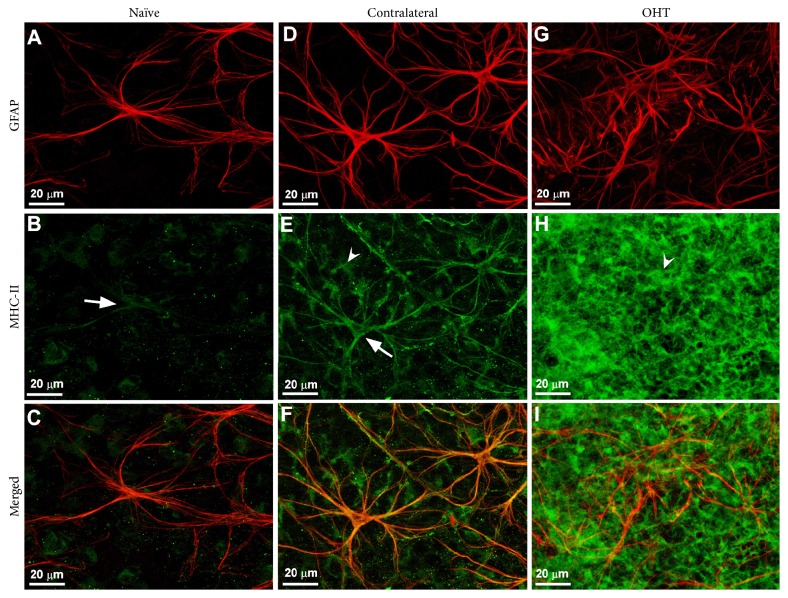
Retinal macroglia in the mouse retina. Retinal whole-mount. Double immunostaining for GFAP (red) and MHC-II (green) after 15 days of laser-induced ocular hypertension. (A)–(C): naïve eyes; (D)–(F): contralateral eyes; (G)–(I): OHT eyes. In contralateral eyes, MHC-II immunoreaction of astrocytes (arrow) and Müller cells (arrowhead) in (E) was increased with respect to naïve eyes (arrow) in (B). In OHT eyes, MHC-II immunoreaction of Müller cells (arrowhead) in (H) was notably upregulated in comparison with contralateral (E). In OHT eyes the Müller cells were GFAP^+^ throughout the retina and appeared as punctate structures between the astrocytes and their radiating processes (G). Fluorescence microscopy and image acquisition using the ApoTome. GFAP: glial fibrillary acidic protein; MHC: major histocompatibility complex; OHT: ocular hypertension (from Figure 10 of [19] with permission).

Notably, when activated, astrocytes express class II MHC and costimulatory molecules on the cell surface, thus stimulating T-cell activation in the CNS (Figure 3) [101]. In an experimental mice model of glaucoma, upregulation of MHC-II expression was found in retinal macroglia in both the hypertensive eye and the contralateral normotensive eye (Figure 4) [19].

Astrocytes and Müller cells can also produce complement proteins (Figure 3). In glaucomatous eyes the presence of C1q in astrocytes and Müller cells lining the inner limiting membrane could be an adaptive mechanism for removing apoptotic RGC [102].

### 3.2. Müller Cell in Retinal Diseases

Considering their strategic location, Müller cells are in position to influence and be influenced by neuronal activity throughout the tissue [15]. Therefore, they are usually one of the first glial cells to detect retinal damage because of their radial distribution, providing a rapid response to any alteration of the retinal microenvironment [75, 103].

Müller cells are more resistant than retinal neurons to various forms of injury such as ischemia, anoxia, hypoglycemia, and elevation in the hydrostatic pressure. This resistance can be attributed to their peculiar energy metabolism and the presence of an energy reserve in the form of glycogen, their high antioxidant content, their capacity to proliferate and regenerate, and the presence of glutamate transporters and glutamine synthetase that rapidly detoxify excess glutamate, among other compounds [75, 103].

In Müller cells, gliosis is characterized by both nonspecific responses, that is, stereotypic alterations independent of the causal stimulus: the increased expression of GFAP and the activation of the extracellular signal-regulated kinases (ERKs) [18]. The upregulation of GFAP is therefore used as a common marker for reactive Müller cells and is so sensitive that it can be used as an indicator of retinal stress, retinal injury, and Müller cell activation [104]. This upregulation of intermediate filaments (GFAP, vimentin, and nestin) seems to be a crucial step for the gliotic response involved in glial scar formation, monocyte infiltration, neurite growth, neovascularization, and cell integration [18, 79].

Practically all retinal diseases are associated with the gliosis of Müller cells. In experimental diabetic retinopathy, glial reactivity is manifested by increased GFAP immunoreactivity and content in both Müller cells and astrocytes [105]. Such increment of GFAP has also been reported in the retinas of patients with nonproliferative diabetic retinopathy [106].

Müller cells are among the first to respond following intraocular pressure increase and it is thought that reactive Müller cells in glaucoma could increase the susceptibility of RGCs to stress signals and contribute to disease progression [75]. In human glaucomatous eyes and in experimental and hereditary animal models of glaucoma, more intense expression of GFAP in Müller cells has been detected in the retina [19, 67, 107–109]. In experimental glaucoma, GFAP upregulation in Müller cells is not restricted to the hypertensive eye but is also detected in the normotensive contralateral eye [19, 110, 111]. In AMD, regions of GFAP upregulation in Müller cells can be involved in drusen formation [112]. Such upregulation can occur early in the course of retinal detachment [113] or in response to degeneration of the retina in a rat model of retinitis pigmentosa [114].

Müller cell gliosis may include the dedifferentiation of the cells into pluripotent retinal progenitor/stem cells. Such dedifferentiation represents a precondition for regenerative processes in the injured retina and for glial-cell proliferation and migration [115]. It has been reported that after retinal detachment Müller cells migrate to the outer retina and undergo mitosis. Some of these displaced Müller cells stop to express Müller cell marker proteins, a feature that has been interpreted as dedifferentiation [116]. As a most important step of this dedifferentiation, the cells reduce the K^+^-conductance of their membrane, particularly the Kir4.1-mediated current, which is generally associated with a mislocation of the Kir4.1 channels in the Müller cell membrane [117]. This mislocation of Kir4.1 protein has been associated with a greater vulnerability of RGCs to ischemic stress [118], which will inflict a severe loss of the functions involved in normal neuron-glia interaction [18]. The alteration of Kir4.1 channels has been described in retinal tissues after retinal blue-light injury, after retinal ischemia, in ocular inflammation, and in diabetic rats [119–122].

**Figure 5:**
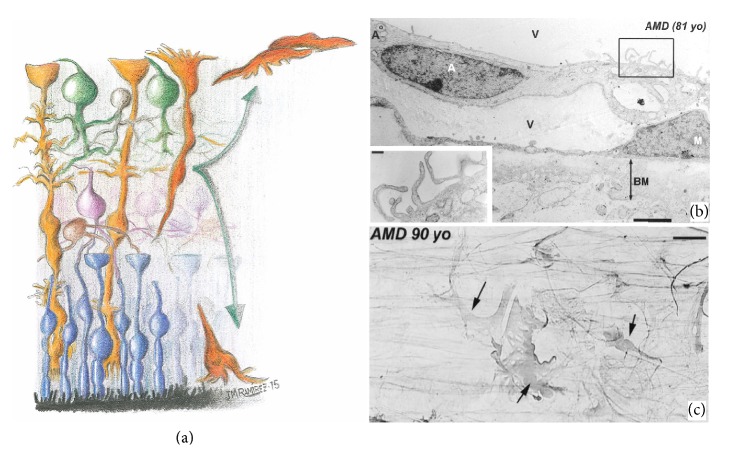
Müller cell gliosis. Schematic drawing illustrating Müller cell proliferative gliosis (a). Transmission electron microscopy of a retina from an 81-year-old patient with age-related macular degeneration (b). Immunoperoxidase anti-GFAP. Retinal whole-mount from a 90-year-old patient with age-related macular degeneration (c). (a) Müller cells reenter into the cell cycle and migrate to the subretinal space and the vitreous humor where they contribute to forming the subretinal membranes and the epiretinal membranes, respectively. (b) Epiretinal astroglial membrane formed by astrocyte and Müller cells located in the vitreous humor. The Müller cells adhere to the vitreous face of the inner limiting membrane. The inset shows the astrocyte microvilli. (c) Glial membrane at the vitreoretinal interphase showing strongly GFAP^+^ Müller cells (arrow). A: astrocyte; BM: basement membrane; M: Müller cells; v: vitreous (schematic drawing modified from [18]; (b) and (c) from Figures 8F and 12A of [47] with permission).

In the retina, proliferative gliosis occurs by reentry into the cell cycle of Muller cells, accompanied by a dramatic alteration in the expression of trophic factor channels and transporters as well as the migration of these cells (Figure 5) [75, 123]. Müller cells react to retinal injury by establishing a glial scar that fills retinal breaks or holes, replacing degenerated neurons and photoreceptors [124]. Glial scars involve the expression of inhibitory molecules on the surface of reactive glial cells, which additionally inhibit regular tissue repair and neuroregeneration, harming the function and structure of retinal neurons [18, 75, 125]. A form of glial scar involves the epiretinal membranes (frequently detected in retinal detachment), AMD, and proliferative diabetic retinopathy [126]. In addition, in AMD, glial membranes constituted by astrocytes and Müller cells have been reported to be located between the vitreous humor and internal limiting membrane of the retina (Figure 5) [48].

Gliosis of Müller cells has both cytoprotective and cytotoxic effects on retinal neurons [127]. After retinal insults, less severe changes in Müller cells have been described as “conservative” or nonproliferative gliosis. In particular, early after injury, Müller cell gliosis is neuroprotective, due to the release of neurotrophic factors and antioxidants which favor neuronal survival and limit the extent of tissue damage [42, 75, 103, 128–130]. After axotomy, excitotoxicity, or experimental glaucoma, Müller cells increase the expression of leukemia inhibitory factor and ciliary neurotrophic factor to promote RCG survival [129, 131–133]. Both in glaucomatous donor eyes in humans and in ocular hypertension in rats, an increased concentration of hypoxia-inducible factor- (HIF-) 1*α* has been detected in Muller cells which induces the expression of neuroprotective factors such as VEGF or EPO [134, 135]. In diabetic retinopathy, Müller glia activation may bolster neuroprotection by releasing angiogenic and neurotrophic factors (Figure 3) in order to protect the retina from hyperglycemia-induced stress [4] and through a mechanism involving ERK1/2 activation [136].

However, the most severe insults provoke yet another level of Müller cell response described as “massive” or proliferative, in which gliosis becomes detrimental to the retinal tissue and increases neuronal death [18]. A possible trigger for the transition from “conservative” to “massive” gliosis is the breakdown of the BRB, augmenting the retinal and vitreal contents of growth factors, cytokines, and inflammatory factors, as well as an infiltration of blood-derived immune cells (Figure 3) [137]. After laser lesions in rat retina, which cause a breakdown of the BRB, the extravasated plasma protein, immunoglobulin G, may further trigger the reactive gliosis of Müller cells [138].

Furthermore hypoxia and hyperglycemia induce the overexpression of angiogenic cytokines and release of matrix metalloproteinases by Müller cells. These metalloproteinases impair the tight junctions via proteolytic degradation of occludin and claudin on retinal endothelial cells and pigment epithelial cells [139].

On the other hand, the excessive and/or prolonged expression of potential protective factors might lead to detrimental neuronal effects. An example is the expression of VEGF, which is highly induced in Müller cells following injury and which has the distinct potential of protecting retinal neurons against apoptosis [140, 141]. However, the excessive and prolonged expression of VEGF by Müller cell as occurs in diabetic retinopathy can lead to retinal inflammation, neovascularization, vascular leakage, and vascular lesion (Figure 3) [142].

Quite comparable observations have been reported concerning the release of NO by reactive Müller glia [75]. High concentrations of nitric oxide can damage neurons [143–145], while lower levels may have beneficial effects, such as the protection of neurons against glutamate excitotoxicity and the decreased retinal ischemia by its vasodilator effect [146]. With respect to glutamate, it has been demonstrated that, in glaucoma, Müller cells lose their capacity to regulate glutamate homeostasis, owing to the reduction in the biosynthesis of glutamate transporter (Figure 3) (GLAST). As a consequence, glutamate accumulates in the intercellular space, provoking neuronal death [147–149].

Another important feature in gliotic Müller cells is their intense crosstalk with cells from the immune system [126]. Molecules from inflammatory cells, platelets, and plasma may activate Müller cells, and these cells may express a wide variety of inflammation- and immune-response-related factors and enzymes such as TNF-*α*, IL, interferon, and ICAM-1 (Figure 3) [75]. Müller cells can mediate direct cytotoxic effects via an intensified expression of TNF-*α* or monocyte chemoattractant protein- (MCP-) 1 [79, 150–152]. Notably, microglial activation induces Müller responses such as an increase in Müller cell-microglia adhesive cell contacts that may guide the intraretinal mobilization of migratory microglia in a radial direction using Müller cell processes as an adhesive scaffold [153].

Under pathological conditions, for example, oxidative stress, inflammatory mediators, retinal laser photocoagulation, or increase in the intraocular pressure, Müller cells show upregulation of MHC class II molecules by acting as antigen-presenting cells (Figures 3 and 4) [19, 75]. Moreover, Müller cells are also able to produce complement proteins (Figure 3) [102].

## 4. Conclusions

In summary, retinal macroglial cells are fundamental for homeostasis of the retinal neurons. These cells form a defensive system of the retina through its complex program of activation termed “the reactive gliosis.” This gliosis can be neuroprotective or neurodegenerative and in the latter case may impair the course of retinal pathologies.
